# Microwave ablation combined with OK-432 induces Th1-type response and specific antitumor immunity in a murine model of breast cancer

**DOI:** 10.1186/s12967-017-1124-9

**Published:** 2017-01-31

**Authors:** Li Li, Wei Wang, Hong Pan, Ge Ma, Xinyi Shi, Hui Xie, Xiaoan Liu, Qiang Ding, Wenbin Zhou, Shui Wang

**Affiliations:** Department of Breast Surgery, the First Affiliated Hospital, Nanjing Medical University, 300 Guangzhou Road, Nanjing, 210029 China

**Keywords:** Microwave ablation, OK-432, Breast cancer, Immunotherapy, T cell

## Abstract

**Background:**

Minimally invasive therapies, such as microwave ablation (MWA), are widely used for the treatment of solid tumors. Previous studies suggest that MWA is feasible for the treatment of small breast cancer, and thermal ablation may induce adaptive antitumor immunity. However, the induced immune responses are mostly weak, and the immunomodulation effects of MWA in breast cancer are unclear. Immunostimulant OK-432 can induce tumor-specific T-cell responses and may augment the immunity induced by MWA.

**Methods:**

We treated 4T1 breast cancer bearing BALB/c mice with MWA, OK-432, MWA plus OK-432, or left without treatment. Survival time was evaluated with the Kaplan–Meyer method comparing survival curves by log-rank test. On day 25 after ablation, surviving mice received tumor rechallenge, and the rechallenged tumor volumes were calculated every 5 days. Immunohistochemistry and flow cytometry were used to evaluate the T-cell immune responses in ablated tissues and spleens. The tumor-specific immunity was assessed by enzyme-linked immunospot assays. Besides, the cytokine patterns were identified from enzyme-linked immunosorbent assay.

**Results:**

Microwave ablation plus OK-432 resulted in longer survival than single treatment and protect most surviving mice from tumor rechallenge. Both local and systemic T-cell responses were induced by MWA and were further enhanced by subsequent administration of OK-432. Moreover, the combination of MWA and OK-432 induced stronger tumor-specific immune responses than MWA alone. In addition, OK-432 and MWA synergistically promoted the production of Th1-type but not Th2-type cytokines, and polarized T-cell responses to Th1-dominant state.

**Conclusions:**

The T-cell immune responses were activated by MWA in breast cancer. Furthermore, the combination of MWA and OK-432 induced Th1-type response and elicited specific antitumor immunity.

## Background

Microwave ablation (MWA) is a relatively new minimally invasive therapy that generates electromagnetic heating and causes focal hyperthermic injury to destroy tumor tissues [1]. MWA shows several advantages over radiofrequency ablation (RFA), the most commonly used thermal ablation technique [2–5], and has proven to be an effective and safe treatment option for liver, lung, and kidney tumors [6]. Our group has previously reported that MWA is feasible for small breast cancer [7].

Tumor debris produced by thermal ablation provide pro-inflammatory signals and serve as tumor antigens to induce adaptive antitumor immunity [1, 8]. Recent study has reported that MWA of osteosarcoma can elicit tumor-specific T-cell immune response in a rat model [9]. T-cell immune responses induced by MWA have also been observed in hepatoma patients [10]. However, the tumor recurrence rate after MWA is similar to that after curative surgical resection [11, 12]. These results indicate that the antitumor immunity induced by MWA is not strong enough to prevent the recurrence of cancers. Thus, additional immunomodulatory strategies are needed to enhance the antitumor immunity.

OK-432, a penicillin-inactivated and lyophilized preparation of a low-virulence strain Su of Streptococcuspyogenes (group A), was approved by Japan administration in 1975 as an immunotherapeutic agent in cancers [13]. Previous studies have reported OK-432 induce pro-inflammatory cytokines to activate T-cell-mediated immunity [14, 15]. It has been reported that OK-432 augment antitumor T-cell response in both animal models and patients [16–19].

Despite growing attention, the immune responses induced by MWA in breast cancer are still unclear. The purpose of this study was to investigate the antitumor immunity against breast cancer induced by MWA and whether these immune responses could be promoted by administration of OK-432.

## Methods

### Cell line

The murine breast cancer cell line 4T1 was obtained from Chinese Academy of Sciences (Shanghai, China). The cells were grown (37 °C incubator with 5% CO_2_) in RPMI 1640 supplemented with 10% fetal bovine serum, 100 μg/mL streptomycin, and 100 unit/mL penicillin. All cells culture reagents were purchased from Invitrogen (Shanghai, China).

### Animal models

5 × 10^4^ 4T1 cells in 100 μL of PBS were injected subcutaneously into the right inguinal mammary fat pads of 6- to 8-week-old female Balb/c mice (Vital River Laboratories, Beijing, China). The mice were euthanized when the tumor diameter exceeded 20 mm in diameter or when they became moribund during the observation period, and the time of euthanization was recorded as the time of mortality. All protocols and studies involving animals were approved by Nanjing Medical University Institutional Animal Care and Use Committee.

### Study design

Animals with established tumors were randomized to four groups. Mice in MWA group were treated with microwave ablation alone. About 30 min after MWA, 1 Klinishe Einheit (KE; i.e., 0.1 mg) OK-432 (Lukang Pharmaceutical, Shandong, China) in 100 μL PBS were injected peritumorally in the combination treatment group, whereas mice treated with MWA alone were injected with 100 μL of PBS. The injection was repeated once 3 days later. Mice in OK-432 group received OK-432 only. No-treatment group served as control.

For rechallenge test, mice that survived for 25 days after MWA and age-matched healthy mice were injected subcutaneously with 2 × 10^4^ 4T1 cells in 100 μL of PBS into the left inguinal mammary fat pads. Subsequently, the sizes of second tumors were measured with calipers every 5 days, and tumor volumes were calculated using the formula 1/2 (Length × Width^2^).

### Microwave ablation

On day 35 after tumor implantation, when tumor size reached 8–10 mm in diameter, tumor-bearing mice were treated with MWA using a microwave generator (ECO-100E, Yigao Microwave Electric Institute, Nanjing, China). Mice were anesthetized with isoflurane (RWD Life Science, Shenzhen, China) inhalation. After disinfected the tumor area with alcohol, a 17-gauge MWA antenna (Yigao Microwave Electric Institute, Nanjing, China) was inserted into the center of the tumor. According to the results of the pre-test, MWA was performed at a power output of 5 W for 3 min to achieve complete ablation of primary tumors. The microwave irradiation frequency is 2450 MHz.

### Immunohistochemical analysis

The removed tumor tissues were fixed in 4% formalin solution and paraffin embedded. Paraffin sections were stained with rat anti-mouse CD4 (4SM95, eBioscience, San Diego, USA) and rat anti-mouse CD8 (4SM15, eBioscience) followed by horseradish peroxidase (HRP)-conjugated goat anti-rat IgG (Santa Cruz Biotechnology, Santa Cruz, USA) and diaminobenzidine visualization (DAB kit, Beyotime, Nanjing, China). Nuclei were counterstained with hematoxylin. The numbers of positive cells were counted in five randomly selected fields at 400-fold magnification. Results from the five areas were averaged and used in the statistical analysis.

### Flow cytometric analysis

The harvested spleen was minced gently with the plunger of a 10 mL syringe and passed through a 70-μm nylon mesh cell strainer (Falcon, New Jersey, USA) to achieve a single cell suspension. Red blood cells were removed by cell lysis buffer (BD Biosciences, San Jose, USA). For intracellular cytokine staining, harvested cells were stimulated with phorbol myristate acetate (PMA), ionomycin, and brefeldin A (Cell Activation Cocktail, Biolegend, San Diego, USA) for 5 h. FITC-anti-CD4 (RM4-5), APC/Cy7-anti-CD8a (53-6.7), and matched isotype control antibodies were purchased from BD Biosciences. PerCP/Cy5.5-anti-CD4 (RM4-5), FITC-anti-IFN-γ (XMG1.2), APC-anti- interleukin (IL)-4 (11B11), and matched isotype control antibodies were purchased from Biolegend. Flow cytometric analysis was performed using a FACS flow cytometer (Beckman Coulter, Miami, USA), and analyzed by Kaluza software (version: 1.1; Beckman Coulter).

### ELISPOT assay

The enzyme-linked immunospot (ELISPOT) assays were performed with Mouse Interferon-γ (IFN-γ) ELISPOT Set (BD Biosciences) according to the manufacturer’s manual. Splenocytes (5 × 10^5^ cells/well) were added to plates precoated with anti-mouse IFN-γ antibody together with Mitomycin C treated 4T1 or CT26 cells (2.5 × 10^5^ cells/well). After cultured at 37 °C with 5% CO_2_ for 48 h, the plates were washed, further incubated with biotinylated detection antibody. Finally, the IFN-γ producing cells were visualized using AEC substrate set (BD Biosciences). The numbers of spots were counted automatically by BioSys BioReader 4000 PRO (Biosys, Karben, Germany).

### ELISA assay

Blood was collected via cardiac puncture into tubes containing EDTA. Plasma was obtained by centrifugation at 1000*g* for 10 min and stored at −70 °C until analysis. Concentrations of IL-2, IL-4, IL-10 and IFN-γ in plasma were measured by enzyme-linked immunosorbent assay (ELISA) using High Sensitivity ELISA Kit (eBiosciences). IL-12p70 and IL-18 were quantified using mouse IL-12p70 Quantikine Reagent Kit (R & D Systems, Minneapolis, USA) and IL-18 Platinum ELISA Kit (eBiosciences) respectively.

### Statistical analysis

The significance of differences between four experimental groups was assessed by one-way ANOVA followed by Student’s *t*-test with Bonferroni’s correction or Dunnett’s test for multiple comparisons. Student’s t-test was used for rechallenge test. Survival was analysed with the Kaplan–Meyer method comparing survival curves by log-rank test. Analyses were done with SPSS software (version: 22; SPSS, Chicago, USA). A P value of less than 0.05 was considered statistically significant.

## Results

### Combination of MWA and OK-432 resulted in prolonged survival and enough immunity against tumor rechallenge

**Fig. 1:**
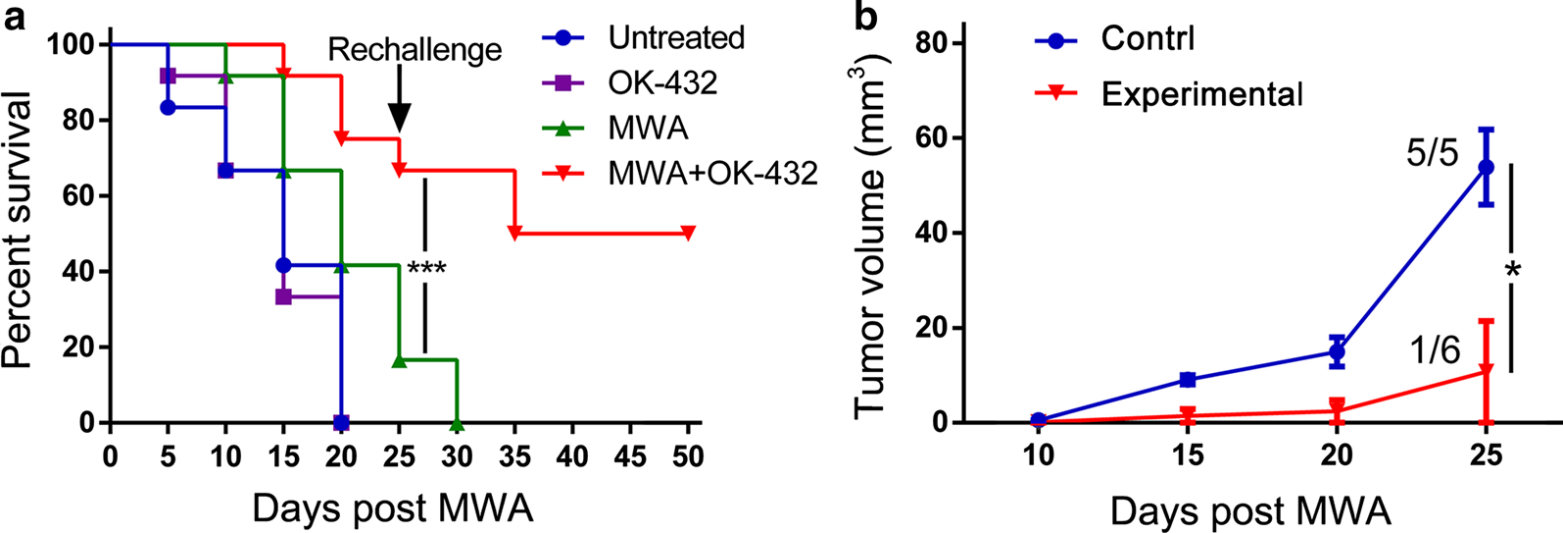
OK-432 enhanced antitumor effects after MWA. **a** Kaplan-Meier curves showing survival of untreated mice and mice treated with OK-432, MWA, and MWA plus OK-432. For the rechallenge test, 2 × 10^4^ 4T1 cells were injected into* left* inguinal mammary fat pads of surviving mice on day 25 after MWA. Age-matched healthy mice served as control (n = 5). **b** Tumor volumes of mice that survived over 10 days after rechallenge were calculated every 5 days (Experimental group: n = 6; Control group: n = 5). On day 25 after rechallenge, 5/5 of control mice and 1/6 of MWA plus OK-432 treated mice developed tumors after rechallenge. Points, mean;* error bars*, SEM. *P < 0.05; ***P < 0.001. One representative experiment out of three is shown

To assess the antitumor effect of MWA, OK-432 and their combination in breast cancer, their abilities to promote survival were compared. Mice implanted orthotopically with 4T1 cells were treated with MWA, OK-432, the combination of MWA and OK-432 or left without treatment. Survival prolongation was observed in MWA treated mice, when compared with mice in OK-432 group and untreated group (P = 0.006 and 0.001, respectively, Fig. 1a), but there was no significant difference in survival between untreated group and OK-432 group. In the combination treatment group, survival was significantly longer than that of MWA, OK-432 and untreated groups (all P < 0.001).

To simulate a clinical recurrence in patients, mice that survived for 25 days after treatment (MWA group: n = 3; MWA plus OK-432 group: n = 8) received reimplantation with 2 × 10^4^ 4T1 cells on the opposite flank. At 10 days after rechallenge, six MWA plus OK-432 treated mice still survivedto allow the evaluation of second tumor, while no mice survived in MWA alone group. Therefore, these survived mice from the combination treatment group (n = 6) were allocated to experimental group and age-matched healthy mice (n = 5) were used as controls. The combination therapy led to complete rejection of the second tumor in 83% (5/6) mice, while all control mice developed palpable tumors after implantation (Fig. 1b). These results indicate that MWA combined with OK-432 prolongs survival, and induces enough antitumor immunity that results in the rejection of rechallenged tumors.

### Local administration of OK-432 promoted CTL, but not Th cell infiltration into MWA treated tumors

**Fig. 2:**
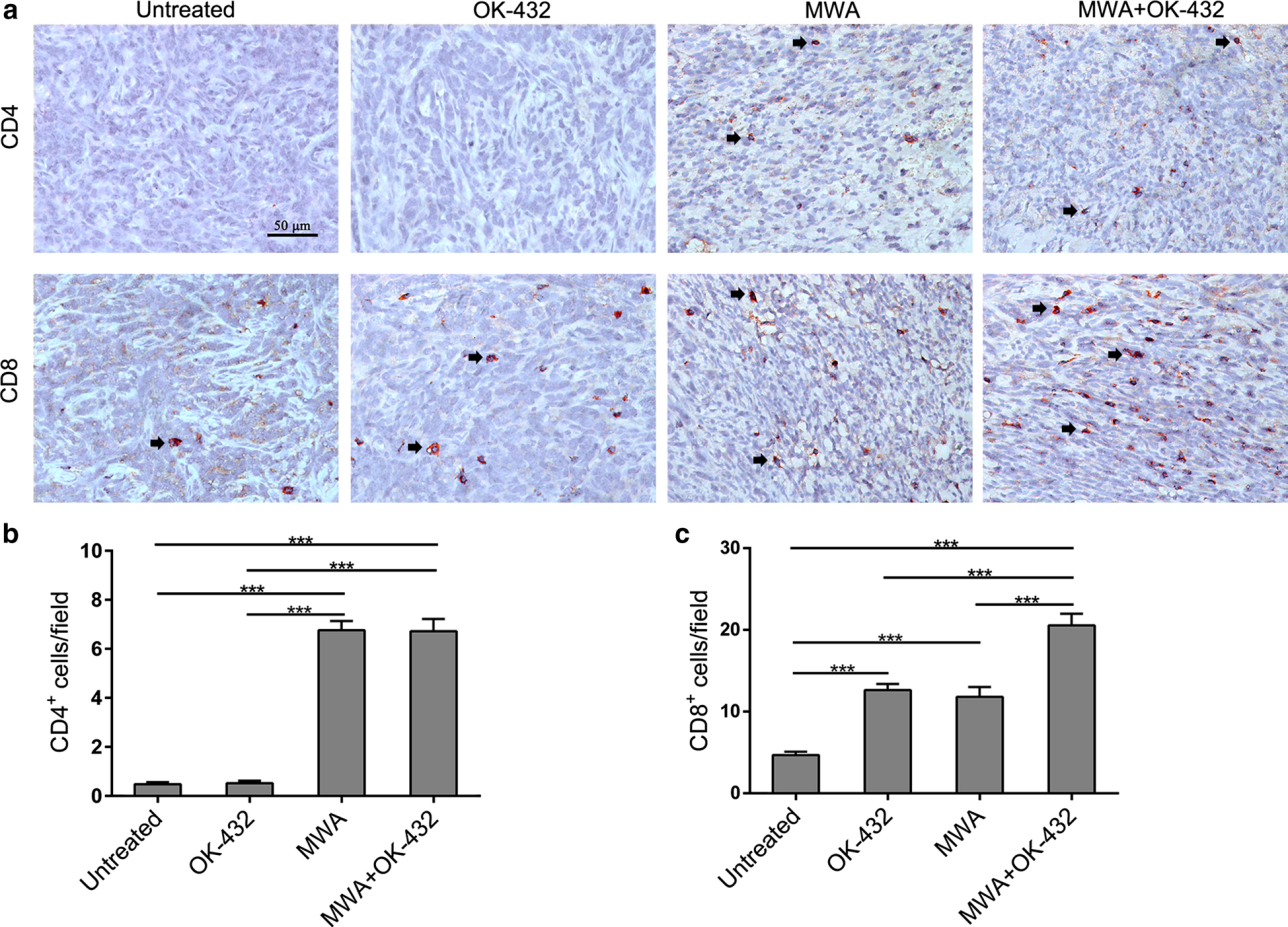
OK-432 increased infiltration of cytotoxic CD8^+^ T-cell into tumors after MWA. **a** representative microphotographs of CD4 and CD8 staining in each group. Immunohistochemical staining was performed on tumor specimens that were harvested 7 days after treatment. Original magnification, ×400. Bar, 50 μm. **b**, **c** Density of CD4^+^ and CD8^+^ cells was determined. Five random areas within a section were chosen and counted at 400-fold magnification. Columns, mean;* error bar*, SEM. ***P < 0.001. Data were pooled from two independent experiments with five mice per group

Given the capacity of OK-432 to activate T-cell responses, the intratumoral infiltrations of CD4^+^ T helper (Th) cell and CD8^+^ cytotoxic T lymphocyte (CTL) were evaluated by immunohistochemistry on day 7 after ablation. The number of intratumoral CD4^+^ T cells was significantly higher in MWA alone and MWA plus OK-432 groups than in no-treatment group (both P < 0.001, Fig. 2b). OK-432 administration showed no impact on CD4^+^ T-cell infiltration into MWA treated or untreated tumors. Either MWA or OK-432 alone increased the number of CD8^+^ T cells infiltrating into the tumors compared with the numbers of these cells infiltrating into untreated tumors (P < 0.001 and =0.001, respectively, Fig. 2c). Moreover, the combination of MWA and OK-432 further enhanced CD8^+^ T-cell infiltration into tumors compared with tumors treated with single treatments (both P < 0.001). These results suggest that OK-432 enhances CTL but not Th cell infiltration induced by MWA.

### OK-432 augmented systemic T-cell immune responses induced by MWA

**Fig. 3:**
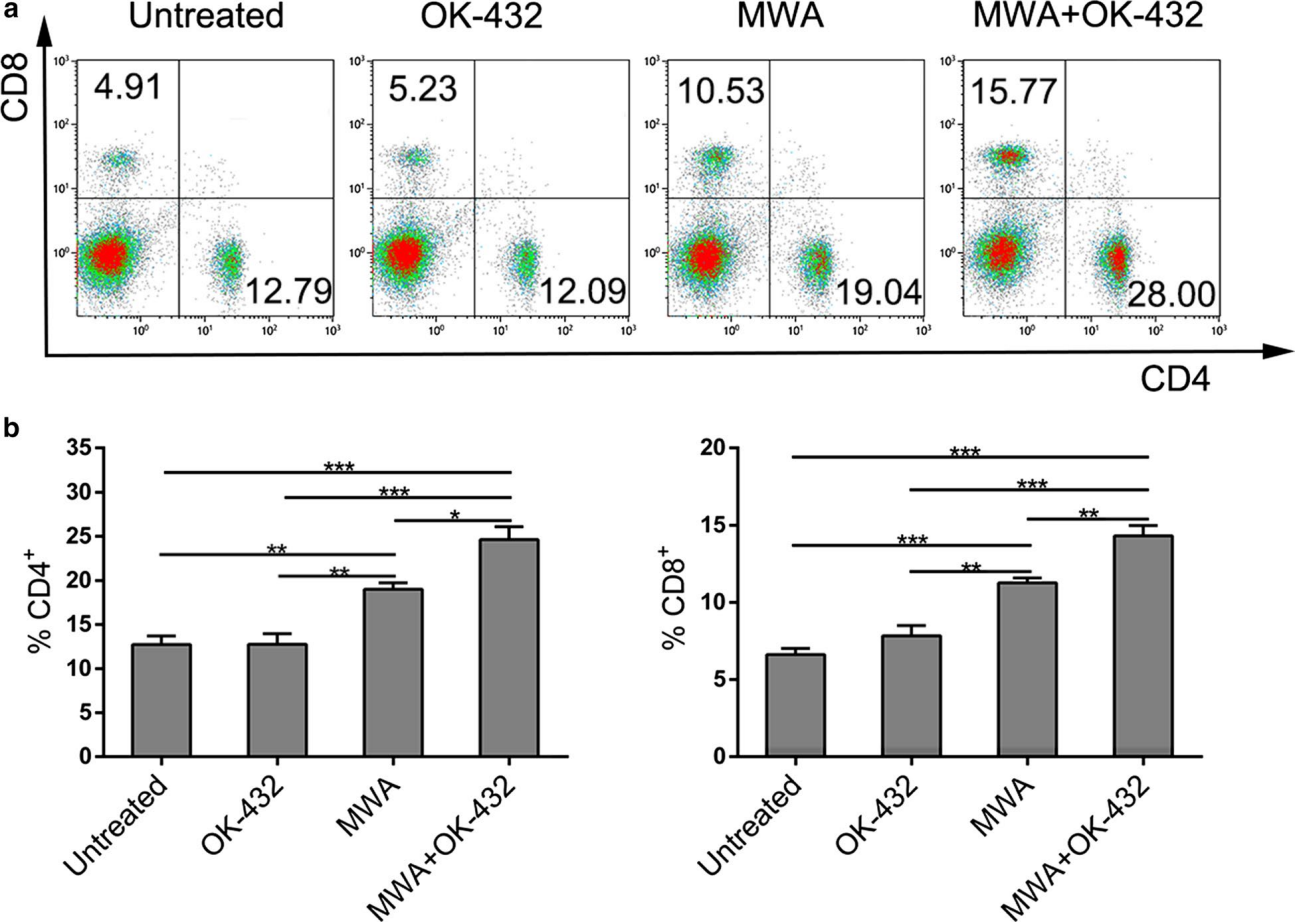
The combination of MWA and OK-432 augmented systemic T-cell responses. Mononuclear cells were prepared from spleen and used for flow cytometric analysis. **a** Representative flow cytometric plots showing CD4^+^ and CD8^+^cells on days 7 after MWA. **b** Percentage of CD4^+^ and CD8^+^ cells in splenocytes on days 7 after MWA. Column, mean; error bars, SEM. *P < 0.05; **P < 0.01; ***P < 0.001; *NS* not significant. Data were pooled from two independent experiments with six mice per group

To determine the impact of MWA combined with OK-432 on peripheral lymphocytes, the levels of CD4^+^ and CD8^+^ T cells in the spleen were analyzed by flow cytometry. On day 7 after ablation, the percentage of splenic CD4^+^ and CD8^+^ T cells of mice treated with MWA alone were significantly increased when compared with those of untreated mice (P = 0.004 and <0.001, respectively, Fig. 3a, b). Subsequent administration of OK-432 after MWA further increased the levels of CD4^+^ and CD8^+^ T cells in the spleen compared with those of MWA alone treated mice (P = 0.011 and =0.004, respectively). However, the proportion of splenic CD4^+^ and CD8^+^ T cells in MWA plus OK-432 group was at similar level to that in MWA alone group 14 days after ablation (data not shown). These data indicate that MWA either alone or in combination with OK-432 induce systemic T-cell responses. The immune responses induced by MWA can be further enhanced by OK-432.

### MWA and OK-432 synergistically induced specific antitumor immunity

**Fig. 4:**
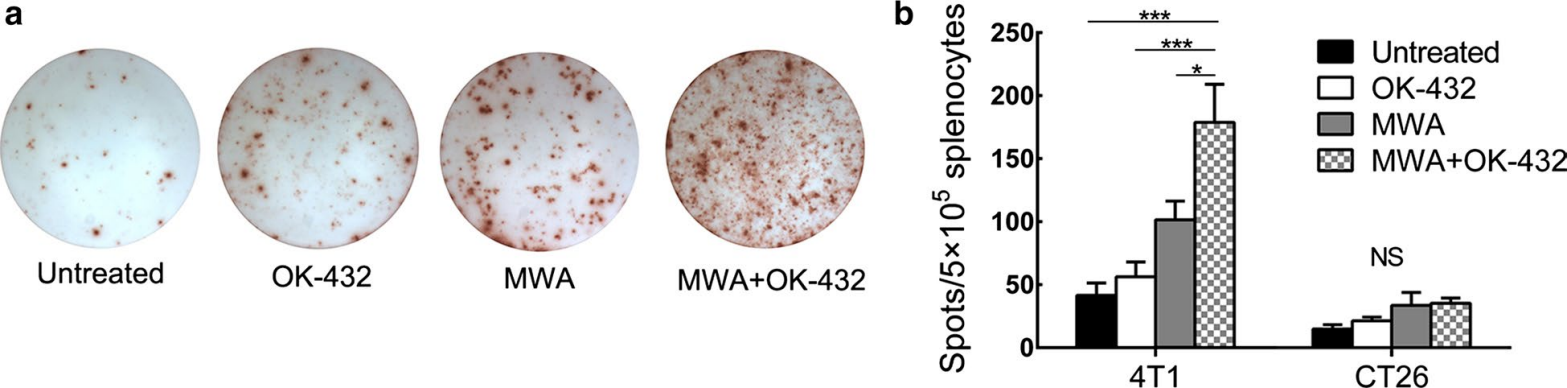
MWA plus OK-432 activated systemic tumor-specific immune responses. IFN-γ secreting cells were analyzed by ELISPOT. Mononuclear cells were prepared from spleen and cultured with 4T1 or CT26 cells for 48 h. **a** Representative ELISPOT images from each group. **b** the ELIPSOT count in each group. Column, mean;* error bars*, SEM. *P < 0.05; ***P < 0.001; *NS* not significant. Data were pooled from two independent experiments with eight mice per group

To further evaluate the specificity of immune response induced by treatments, IFN-γ ELISPOT assays were performed using splenocytes from mice 7 days after ablation. As shown in Fig. 4, the number of 4T1 specific IFN-γ secreting cells was 4- to 5- fold higher in MWA plus OK-432 treated mice compared with that in untreated mice (P < 0.001), while the effect of MWA or OK-432 alone did not meet a significant level of different values compared with no-treatment. The combination treatment group also had higher number of 4T1 specific IFN-γ secreting cells compared with MWA or OK-432 alone groups (P = 0.031 and <0.001, respectively). When the splenocytes stimulated with irrelevant CT26 colon cancer cells, there was no significant difference between groups. These results indicate that MWA and OK-432 can synergistically elicit tumor-specific immune responses.

### Treatment with MWA plus OK-432 resulted in Th1 dominant immunity

**Fig. 5:**
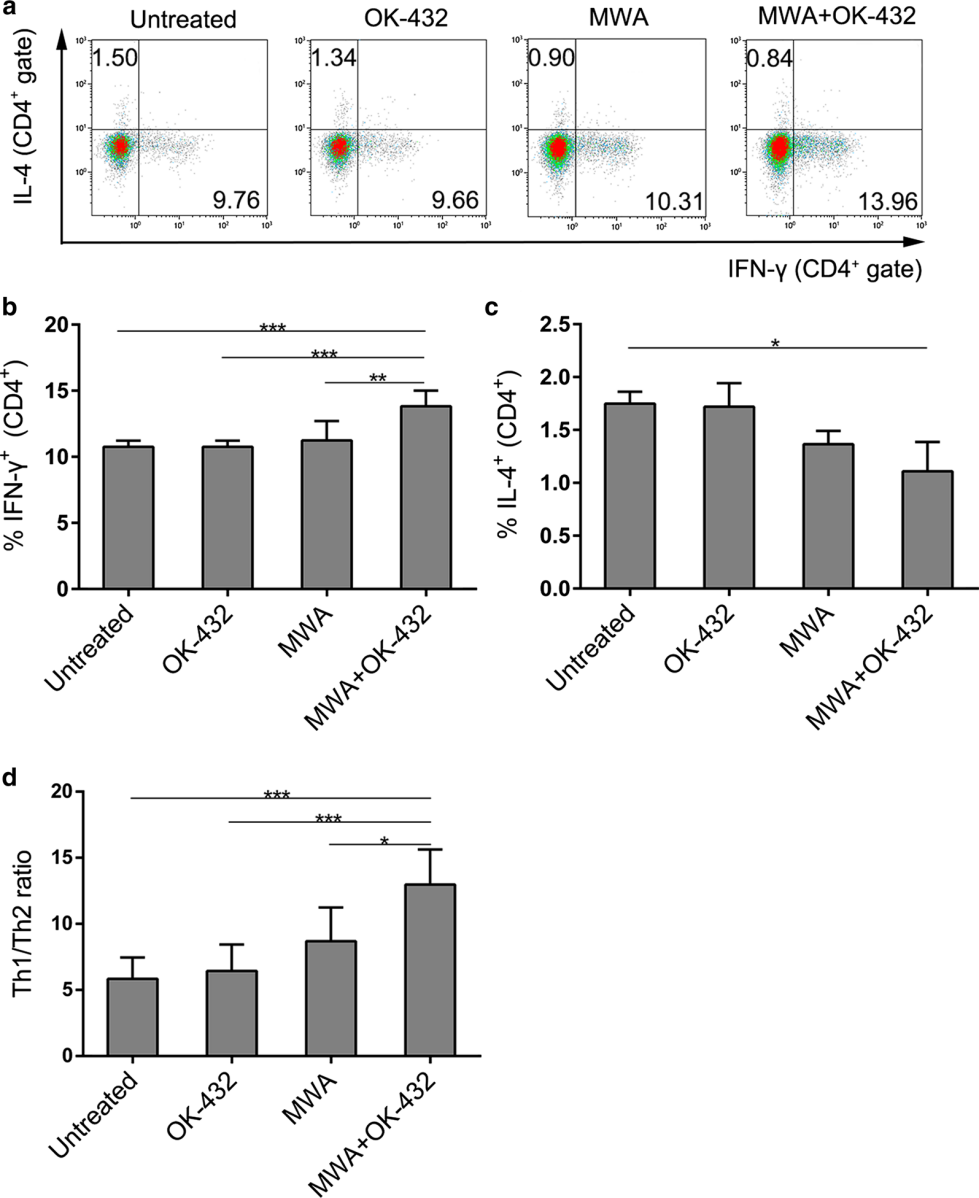
MWA plus OK-432 polarized T-cell responses to Th1 dominance. Mononuclear cells were prepared from spleen and stimulated with PMA, ionomycin, and brefeldin A for 5 h before flow cytometric analysis. **a** Representative flow *cytometric plots* showing CD4^+^ IFN-γ^+^ and CD4^+^ IL-4^+^ cells on days 7 after MWA. **b**, **c**, the percentage of IFN-γ secreting Th1 and IL-4 secreting Th2 cells in CD4^+^ Th cells. **d** The ratio of Th1 to Th2 cells was calculated for each mouse. Column, mean;* error bars*, SEM. *P < 0.05; **P < 0.01; ***P < 0.001. Data were pooled from two independent experiments with five mice per group

Since either OK-432 or thermal ablation has been reported to induce Th1 but not Th2-type immune response [14, 15, 20]. We hypothesized that MWA plus OK-432 would synergistically polarized Th1/Th2 balance to Th1 dominance. To prove this point, Th1 and Th2-type responses were evaluated according to the production of cytokines by CD4^+^ T cells on day 7 after ablation. MWA plus OK-432 treated mice had higher percentage of IFN-γ producing Th1 cells in the spleen compared with MWA or OK-432 alone treated mice (P = 0.004 and <0.001, respectively, Fig. 5a, b). In contrast, the percentage of IL-4 producing Th2 cells was lower in the combination treatment group compared with untreated group (P = 0.05, Fig. 5a, c). Neither MWA nor OK-432 alone affected Th1 or Th2 cell polarization. Therefore, the combination treatment group showed markedly increased Th1 to Th2 ratio compared with MWA or OK-432 alone groups (P = 0.022 and <0.001, respectively, Fig. 5d). These data suggest that although MWA with/without OK-432 enhance CD4^+^ Th cell response, only the combination of MWA and OK-432 can induce a shift of Th1/Th2 balance toward Th1 dominant state.

### Induction of Th1-type cytokines by OK-432 administration after MWA

**Fig. 6:**
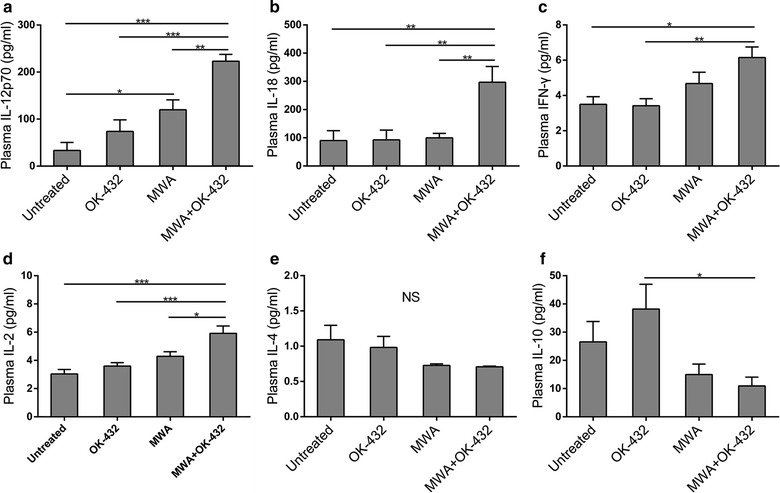
MWA combined with OK-432 induced Th1-type but not Th2-type cytokines. Blood was collected on day 7 after MWA and plasma was isolated as indicated. Levels of cytokines were measured by ELISA. **a**–**f** Plasma concentrations of IL-12p70, IL-18, IFN-γ, IL-2, IL-4, and IL-10 in each group. Column, mean;* error bars*, SEM. *P < 0.05; **P < 0.01; ***P < 0.001; *NS* not significant. Data were pooled from two independent experiments with six or seven mice per group

To further evaluated the levels of Th1 and Th2 related cytokines, plasma concentrations of multiple cytokines were measured as indicated. The levels of pro-inflammatory Th1-type cytokines IL-18 and IL-2 in MWA plus OK-432 group were significantly higher than those in MWA, OK-432 and no-treatment groups (IL-18: P = 0.007, 0.005, and 0.005, respectively; IL-2: P = 0.027, <0.001, and <0.001, respectively; Fig. 6b, d). Another Th1 related cytokine IL-12 was upregulated in MWA alone treated mice compared to untreated mice (P = 0.033, Fig. 6a) and it was further enhanced by combining with OK-432 (P = 0.008). In addition, IFN-γ concentration in the combination treatment group was higher than those in OK-432 alone and no-treatment groups (P = 0.010 and 0.013, respectively, Fig. 6c). Interestingly, OK-432 alone elevated the anti-inflammatory Th2-type cytokine IL-10, which was reduced when combined with MWA (P = 0.033, Fig. 6f). Plasma level of IL-4 was not significantly affected by any of these treatments (Fig. 6e). These data indicate that the combination treatment enhance the production of Th1-type but not Th2-type cytokines.

## Discussion

As a minimally invasive technique, MWA of breast cancer may achieve complete tumor ablation with less surgical complications, better cosmetic outcomes, shorter recovery times, and reduced health-care costs [1]. Our prior study has shown that MWA was a feasible approach for small breast cancer [7]. In addition to hyperthermic injury, the immunological effect induced by thermal ablation may contribute another mechanism of tumor cell death and destruction [1].

During tumor destruction caused by thermal ablation, intracellular tumor-specific antigens are released and captured by antigen-presenting cells in lymphatic tissue [8, 21]. These antigens are processed and presented to T cells to activate antitumor immunity [22, 23]. Recent research has shown that both local and systemic T-cell responses were elicited by MWA alone in hepatoma and osteosarcoma [9, 10]. Although MWA induced T-cell responses in breast cancer were firstly reported by us, the median survival of MWA treated mice was only 5 days longer than that of untreated mice. Previous study has reported that T-cell response induced by MWA is not enough to protect patients from relapse, while immunocyte infiltration extent in ablated tissue is inversely correlated with overall survival and local recurrence rate [10]. Thus, combining MWA with immune adjuvants to enhance antitumor immune response may achieve additional clinical benefit.

Several studies have explored the combination strategies to augment antitumor immunity by administration of immunostimulant, such as IL-2, IFN-β, and staphylococcal enterotoxin C (SEC) [24–26]. Nevertheless, these adjuvants are still under preclinical development. In contrast, OK-432 has been used as immuno-oncology agents in clinical practice for over 40 years [13]. Previous studies have reported the ability of OK-432 to improve the survival of patients after resection of lung or gastric cancers [27, 28]. Our data showed that local injection of OK-432 after MWA markedly prolonged the survival of mice and protected most surviving mice from tumor rechallenge, suggesting that this combination therapy could provide augmented clinical benefit and may prevent tumor recurrence. Similar to the results of Behm et al and Iida et al who used RFA in animal models [20, 29], we observed increased number of T cells infiltrated into ablated tissues. Moreover, we showed that OK-432 could further enhance CTL rather than Th cell infiltration that induced by MWA. In addition, our findings showed that OK-432 plus MWA not only promote peripheral T-cell responses, but also synergistically elicit systemic tumor-specific immunity. This augmented antitumor immunity might result in the tumor rejection in rechallenge test.

Next, we explored the role of CD4^+^ Th cell upon this synergistic effect on immune system. Th cell can be classified generally into two predominant subtypes as Th1 and Th2 cell, which can cross-inhibit each other [30]. Although Th2 cell has been reported to promote the recruitment of eosinophils and macrophages into the tumor microenvironment [31], it appears to contribute to tumor progression in another research [32]. On the other hand, Th1 cell promotes durable tumor-specific CTL responses and induces strong immunological memory against tumor rechallenge [31, 33]. In this study, we found that Th1 to Th2 ratio in the combination treatment group was significantly higher than that of other groups. These data may suggest that skewing of Th1/Th2 balance toward Th1-dominated immunity is responsible for the augmented T-cell responses that observed in the combination treatment group. Additional, plasma levels of Th1-type cytokines, including IL-12, IL-18, IL-2, and IFN-γ, were increased in mice treated with MWA plus OK-432, whereas Th2-type cytokines were not affected. As previously mentioned, OK-432 could augment the production of IL-12 and IL-18 [14, 15]. These two cytokines were shown to synergistically induce T cells to differentiate into Th1 cells [34, 35], and stimulate IFN-γ production from Th1 cells [36]. Th1 cell secreting IL-2 is crucial to the expansion of CD8^+^ T cells and is particularly important for the functional maturation of activated T cells [37, 38]. These results may indicate that combining OK-432 with MWA could produce various Th1-type cytokines to activate Th1-type response, and this process might be potentially an important mechanism underlying the synergistic effect of this combination therapy.

Several limitations in our study should be noted. First, the optimal dose or schedule of OK-432 has yet to be determined. Based on the data from previous animal experiments [39], mice in our study received two doses of OK-432 (1 KE per mouse) given 3 days apart. However, the synergistic effect on Th cell was not observed 14 days after MWA. Thus, a longer duration of OK-432 treatment might provide greater therapeutic benefits. Second, the progression and immune responses in metastatic lesions were not evaluated when primary tumors were ablated. Moreover, the antitumor effects of Th cell and CTL could be further demonstrated through additional experiments, such as CD4^+^ or CD8^+^ T cell depletion. Also, the changes in regulatory T cell and myeloid-derived suppressor cell were not examined to determine whether MWA affects the tumor immunosuppressive environments. Finally, antitumor immunity may vary widely with different tumor host systems. Thus, the immunological effect of MWA and OK-432 should be validated in other animal models and humans.

In conclusion, the present study showed that MWA in breast cancer could elicit T-cell infiltration and systemic T-cell responses. Subsequent administration of OK-432 induced multiple Th1-type cytokines and polarized T-cell responses to Th1 dominance. Furthermore, the combination treatment augmented both local and systemic T-cell responses, and synergistically elicited strong tumor-specific immune responses. Lastly, MWA plus OK-432 prolonged the survival of 4T1 breast cancer bearing mice and resulted in complete rejection of tumor rechallenge in most surviving animals. These data together indicate that the combination of OK-432 and MWA is a potent activator of antitumor immunity. Considering the feasibility and efficiency of either MWA or OK-432 in the treatment of tumors [6, 7, 28], this combination offers a novel treatment option for breast cancer.

## Conclusions

Thermal ablation was previously reported to elicit antitumor immunity in several solid tumors, but little is known about the immunomodulation activated by microwave ablation in breast cancer. In this study, we showed that T-cell responses was induced by microwave ablation in a murine breast cancer model, and the combination of microwave ablation and immunostimulant OK-432 synergistically polarized the T-cell responses to Th1 dominance and augmented specific antitumor immunity. Considering the feasibility and efficiency of either MWA or OK-432 in the treatment of solid tumors, the combination of microwave ablation and OK-432 might be a promising treatment option for breast cancer.

## Abbreviations

MWA: microwave ablation
RFA: radiofrequency ablation
KE: Klinishe Einheit
HRP: horseradish peroxidase
PMA: phorbol myristate acetate
IL: interleukin
ELISPOT: enzyme-linked immunospot
IFN-γ: interferon-γ
ELISA: enzyme-linked immunosorbent assay
Th: T helper
CTL: cytotoxic T lymphocyte
SEC: staphylococcal enterotoxin C
